# Remarkable developments in our performance

**DOI:** 10.1080/03009734.2020.1830579

**Published:** 2020-10-12

**Authors:** Arne Andersson, Joey Lau Börjesson, Kerstin Westermark

**Affiliations:** a Editor of Upsala Journal of Medical Sciences;; b Editorial assistant;; c Chair of Upsala Medical Society

Most certainly, this is the last time you will hold a newly printed issue of *Upsala Journal of Medical Sciences* (*UJMS*) in your hands. For quite a long time we have discussed whether we should join the present trend in scholarly publishing, i.e. terminating the printing of scientific journals. In our case, it will mean an interruption of a 155-year-old tradition (1). It should, however, save us money since both printing and distribution costs have increased lately. An introduction of article processing charges (APCs) paid by the authors might have solved these financial problems. We are, however, not particularly fond of that alternative and wanted to avoid that type of burden on scholarly publishing. You might remember last year’s editorial dwelling on cOAlition S and how that could affect our journal (2,3). That combination of Gold Open Access publication at no cost for the authors is unique and should attract manuscripts from all over the world with, we hope, interesting research news. On top of that, one should not underestimate the impact of a farewell to the printing era on our aim to produce a sustainable journal.

All in all, this has made us look for an alternative publishing process. Last February, the editors of our journal and the *Scandinavian Journal of Urology* gathered editors from medical journals edited in Sweden for a 1-day conference at Svenska Läkaresällskapet (i.e. the Swedish Medical Society) in Stockholm. Amongst the speakers a small company had been invited – Open Academia – run by two people with great experience in scholar publishing who at present serve nine other journals. They offer the exact key services we need and, what’s more, at a modest price. Most importantly, we can retain control over the design and maintenance of our journal website and layout. Moreover, finances are based on a cost-per-published-page model. We are very much looking forward to this collaboration. There will be a lot of work but also opportunities to give both our journal and our website a fresh look.

**Figure 1.**
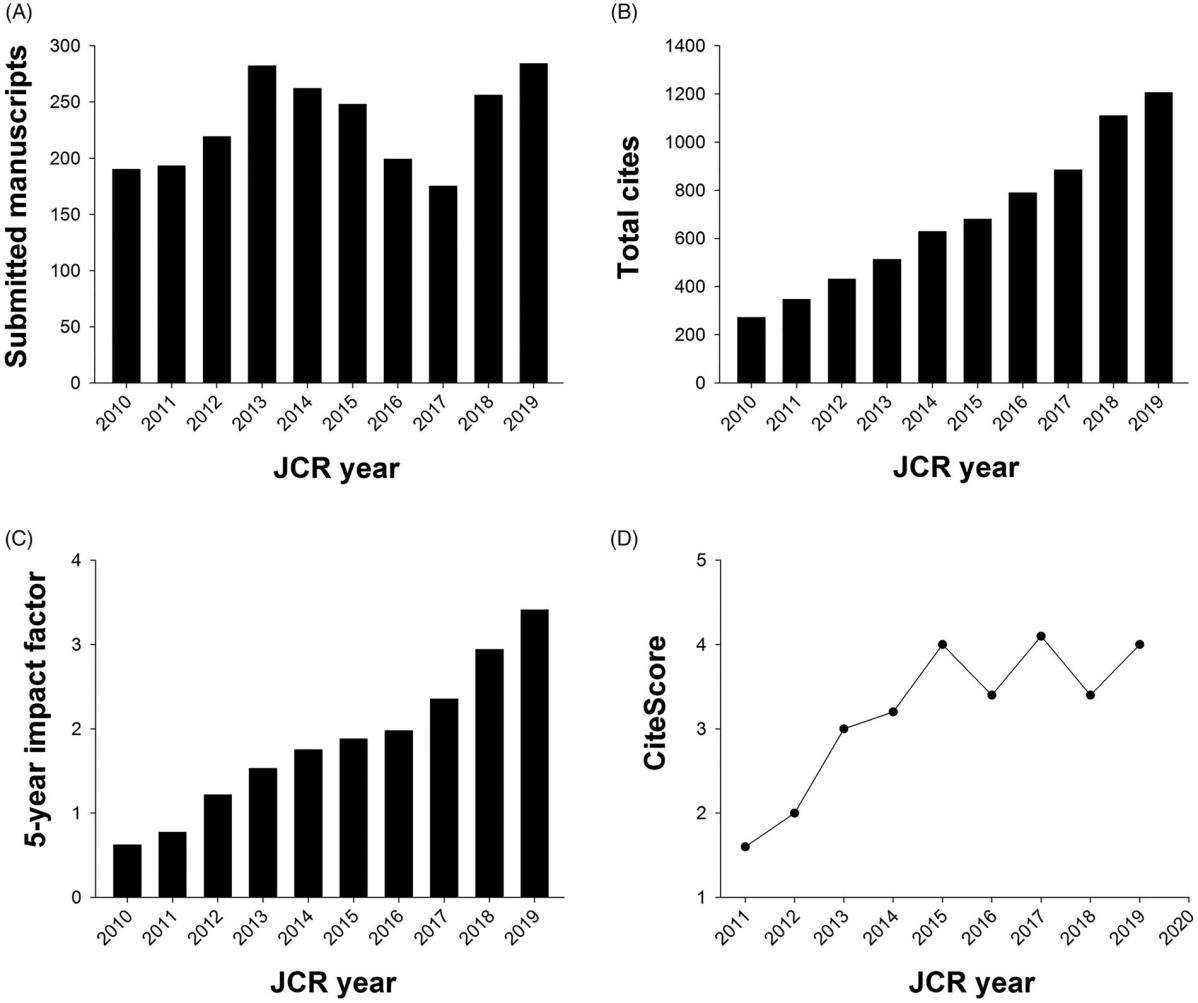
Metrics of UJMS over the latest 10 years.

At this point in time, it might be a good idea to look back to see what we have achieved together with Taylor & Francis (T&F) over the last 10 years. The number of submitted manuscripts has doubled (Figure 1(A)), the number of total cites in the Clarivate statistics has increased by a factor of four (Figure 1(B)), and, finally, the 5-year impact factor has now passed the 3.0 level (Figure 1(C)). A couple of comments on these figures might be of interest. First of all, the lack of an increase in the submission figure last year was due to a maintenance failure of the submission portal during a 5-month period. This year the figure will be close to 400 submitted manuscripts. Combining this figure with the number of published papers per year, 40–50 per volume, gives a rejection rate of about 80–90%. This might be the back of the coin. Naturally, the lack of APCs is of great interest to researchers in financially disadvantaged research environments. Numbers of total cites – perhaps the most relevant citation figure published – have also increased over the years from one per day to more than three at present. Finally, this year’s impact factor value – we prefer to show the 5-year value since it is the most reliable figure for small journals – has passed another whole number level for the first time.

In our editorial 2 years ago we elaborated on the introduction of a new bibliometric scoring system introduced by Elsevier – the so-called CiteScore (4). Since then, they have changed the calculation procedure. Rather than using citation figures for the last 3 years, they now use the last 4 years. For *UJMS* it has meant an obvious change for the better. We are still placed just above the top 90th percentile amongst journals in ‘General medicine’, but our score value has more than doubled over the last 10 years (Figure 1(D)).

We are grateful for this opportunity to thank some of our collaborators at T&F – Håkan Pårup, Therese Granlund, and Sarah Hands – all of whom have contributed extensively over this 10-year period. One major problem for all journals of our size and reputation is finding referees for the submitted papers. We truly pay tribute to all of you who have graciously offered your time to read our manuscripts. Without better support for this system – yes, it might be regarded as old-fashioned, but still there is no better alternative – it might all collapse and be handed over to strictly commercial interests. This is an issue, very much emphasized in the summing-up document of the editor conference mentioned above.

So, although this is the last issue of the journal produced on paper in the classical way, we plan to continue to publish four issues a year as before. When each issue has been finalized it will be sent as a PDF file to all members of the Upsala Medical Society by e-mail. You will be able to glance through it as you can do with many of our daily journals nowadays or indeed even print it out. In the latter case, you will be able to save it on your bookshelf in the time-honoured way for simple and reliable consultation whenever you like.
